# Everybody's Page

**Published:** 1891-09-19

**Authors:** 


### EVERYBODY'S PAGE.

SOME critics, have it seems, taken Mr. Rudyard Kipling to task for want of accuracy in his pathology. This seems hypercritical; a novelist is only a specialist in so far as he is a novelist.

SPORTSMEN are always on the look out for a new animal to hunt, and Frenchmen have now joined in the pursuit of the investigation of ghosts, haunted houses, &c. The results of their efforts in this direction will be recorded in the *Annales des Sciences Psychiques.*

THE merits of ether and chloroform as anæsthetics formed the subject of a recent discussion before the Ohio State Medical Society, which, as is usual in such cases, elicited much conflict of opinion, though the majority expressed want of confidence in chloroform. One doctor, however, expressed unabated confidence in it, stating that he invariably gave whisky some time before administering the anæsthetic.

IT is alleged that in India and in Europe the motives for suicide are not similar. In the former country they are anger, disease, and grief, grief being the chief cause of suicide among women; whereas in Europe the motives are mainly alcoholism, love, misery, and fear of punishment. It is curious that the proportion of suicides amongst Parsees is great when compared with the smallness of the community.

A CRITIC, writing in the *American Druggist* of the twenty-sixth annual Report of the Alumni Association of Philadelphia, observes with regard to one of the rules which appear in the report as to the use of the eyes, that the Association does not appear to have regarded especially the one which reads, "Read only good-sized, black letters, printed on white paper, sufficiently thick to prevent confusion from the print on the opposite side."

NON-PROFESSIONAL readers, who wish to pick up some knowledge on the subjects of heredity, health, and personal beauty, will find Dr. John Shoemaker's work useful and practical.

CHLORIDE of zinc is stated to be a good remedy for perspiration of the feet, if applied in a five per cent solution after washing the feet in tepid water. A sponge can be used for applying the solution, which should be washed off a few minutes after application.

IT is stated that the *régie* system which prevails in France in the case of tobacco, will shortly be adopted by the Dutch-Indian Government for the conduct of the opium traffic. Hitherto it has been the practice to sell by auction the right to retail opium for smoking purposes. Needless to say, this practice has led to abuses both numerous and great.

ADVERTISING proprietary medicines in daily and other journals in the United States has reached such a pitch as to call forth energetic protests from the medical profession. The evil is such that many a patient has been hastened into the grave by adopting some method of treatment which has been recommended in the newspaper advertisements.

ANYONE who receives a large correspondence must frequently have been exasperated at the utter illegibility of some of the signatures at the foot of letters. People who have had a fair education are often the greatest sinners in this respect, and illegibility of writing in general appears to have become such a general disease in Austria that the Minister of the Interior has issued an ordinance that the burgomasters of all communes must see that every medical prescription is clearly written in all its parts, so that there may be no doubt as to the dose or the signature.

